# Pool Boiling Heat Transfer Characteristics of New and Recycled Alumina Nanofluids

**DOI:** 10.3390/nano13061040

**Published:** 2023-03-14

**Authors:** Wagd Ajeeb, S. M. Sohel Murshed

**Affiliations:** IDMEC, Department of Mechanical Engineering, Instituto Superior Técnico, University of Lisbon, 1049-001 Lisbon, Portugal

**Keywords:** pool boiling, critical heat flux, burnout heat flux, nanofluids, recycling

## Abstract

This paper reports an experimental investigation of the heat transfer features of new and recycled Alumina (Al_2_O_3_) nanofluids (NFs) in the pool boiling (PB) system. The mixture of ethylene glycol (EG) and distilled water (DW) is selected as the base fluid (BF), and NFs samples of two low concentrations (0.01 and 0.05 vol.%) of Al_2_O_3_ nanoparticles were prepared. Furthermore, the characteristics of the prepared NFs are evaluated to investigate the heat transfer performance as well as the reusability of the NFs for long-term applications and recycling consideration. Although there have been a large number of boiling studies with NFs, the current study is the first of its kind that addresses the mentioned operation conditions of recycling NF samples. The results are compared with the relevant BF in terms of properties, critical heat flux (CHF), burnout heat flux (BHF), and the convection coefficient of the Al_2_O_3_ NFs in the PB system. The results showed good enhancements in both CHF and BHF of these NFs yielding up to 60% and 54% for BHF at 0.05 vol.%, respectively. The reusage of the previously used (recycled) Al_2_O_3_ NF showed a considerable increase in heat transfer performance compared to base fluids but slightly lower than the newly prepared one. The results of the reused nanofluids demonstrate the great prospects of their recyclability in heat transfer systems and processes such as in pool boiling.

## 1. Introduction

In the last decades, researchers and industries have paid tremendous attention to enhancing the heat exchange process and thermal performance in numerous applications that contain cooling or heating systems, such as electronics, chemical production, and energy systems. New designs of heat exchangers are already available, providing smaller dimensions and higher heat transfer capacity in the devices [1,2]. Phase-change-based heat transfer systems, such as heat pipes in all scales (small to large), are of tremendous interest due to their potential for improved thermal management. Boiling is a phase change heat transfer process that has been employed among the most effective heat-exchanging techniques in many thermal engineering applications, particularly in nuclear systems. Boiling demonstrates a complicated phenomenon involving anarchic bubble dynamics [3]. However, the development of the techniques used in the boiling method was limited due to employing conventional thermal fluids despite high critical heat flux (CHF) and high pool boiling heat transfer coefficients (PBHTC). It becomes clear that the enhanced heat transfer values of the conventional thermal fluids are beneficial to design compact and efficient heating and cooling systems that are used in many industrial applications such as refrigeration, cooling of electronics devices, nuclear and chemical reactors, power generation and heat exchangers [4,5,6,7,8]. Since the coining of NFs in early 1990, it was found that adding nanoparticles (NP) to conventional thermal fluids increases their thermal properties and features, such as thermal conductivity [9,10] and convective heat transfer coefficients [11,12,13], and also enhances their heat exchange capacity in conventional heat exchangers and heat pipes [11,12,13,14] and boiling systems [15]. In addition, this NP (Al_2_O_3_) was found to show good dispersion into the BFs which led to important attention in numerous thermal applications [16,17]. Using NFs to increase the CHF of the conventional fluids in the heat-exchanging process of PB can lead to improved cooling of high heat generating devices (such as high heat generating electronics) and systems providing protection and safety by working with low temperatures during the operation [18]. Since the primary studies showed a considerable increase in boiling performance of NFs [19,20,21], recently some investigations have been reported to explore the performance of NFs in boiling systems [15,18]. A study was conducted by Pare and Ghosh [22] on the transient properties of Al_2_O_3_/DW NFs in nucleate PB and they reported a heat transfer drop around 90% and an increase of nanoparticle deposition of 50% to 300% with increasing heat flux, NF concentrations, and boiling durations. Another study by Wang et al. [23] showed Al_2_O_3_ NFs to improve the CHF of PB in In-vessel Retention. The results indicated a stable enhancement in CHF during the boiling period. Additionally, Mori et al. [24] suggested adding a plate of honeycomb on the heating area while using TiO_2_ NF in a saturated PB, and a significant enhancement of CHF was observed by maximum implementation of the new technique with NFs. In addition, TiO_2_ NF has been investigated in PB by Hadži’ et al. [25] and an improvement in CHT of NF at 0.1 wt.% of NPs compared to DW was reported. The study was supported with SEM images before and after the exposure of NF to boiling, and a wettable layer of TiO_2_ NPs on the surface was observed and specified as a key reason for the enhanced thermal performance of the PB. Moreover, a study by Dadjoo et al. [26] investigated the performance of PB with SiO_2_ NF and an increase in the CHF was found by the inclination of the heater surface angle from 0° to 90°. On another hand, an experimental study by Ma et al. [27] investigated graphene-silver NFs for three concentrations of particles (0.001, 0.002, 0.003 wt.%) for thermal application in PB. A maximum enhancement in the CHF of about 52.31% was obtained for the NPs concentration of 0.001% more than the BF.

Although some studies investigated the heat transfer performance of some types of NFs in PB [4], the boiling (pool) performance and mechanisms of NFs are not yet conclusive and well-understood. Therefore, further investigations employing various NFs in PB systems under various conditions are still of great importance. In addition, NFs in boiling systems have been limitedly investigated in the literature, and the presented data of PBHTC and CHF were inconsistent [28]. However, investigations into the thermal behaviour of certain NFs are still needed, particularly using low NP concentrations. Furthermore, one of the main challenges of applying NFs is their shelf life. NF shelf life is an important point that researchers have missed in most of the investigations on NFs so far. The latter raises the question of whether NFs are worth recycling even for the same purpose or if they should be thrown away as one-time materials. Nevertheless, none of the studies has discussed the reuse (at least a second time) of NFs and the effects of the operation conditions on their features to be used again. Using NFs one time and then throwing them away is neither economically efficient nor environmentally friendly. Therefore, this study focuses on studying the features of NFs on the first use and the second use (after around two months of the first use) in bool boiling applications. Thus, an experimental investigation is carried out on PB of EG/DW mixture-based Al_2_O_3_ NFs with consideration for the reusability of the NF samples for the same application.

## 2. Preparation and NF Samples

**Figure 1:**
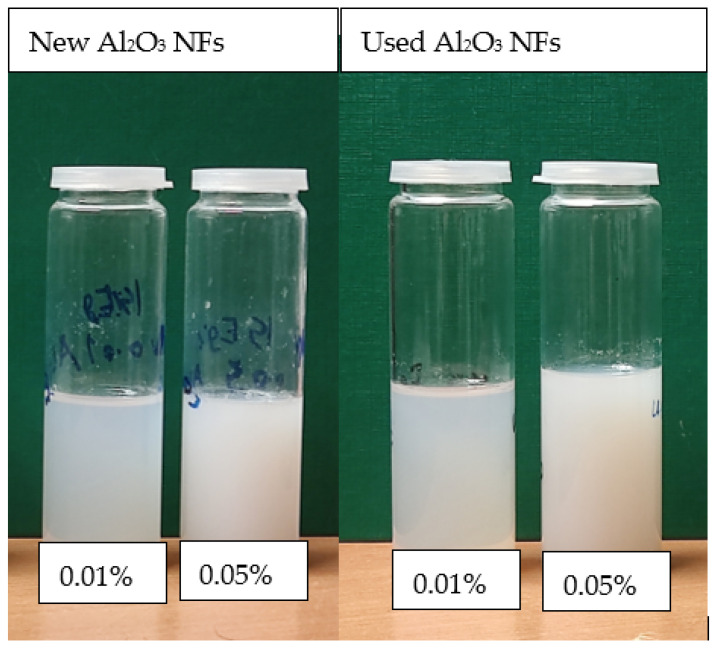
NF samples of two different concentrations before use in boiling experiments.

The NF samples are prepared using two volumetric concentrations (0.01 and 0.05 vol.%) of Al_2_O_3_ NPs (average particle size: 70 nm, Purity: 99% as provided by the supplier Alfa Aesar, Lancaster, UK, and the mixture of 15%EG/85%DW (*v/v*) as a BF). EG/DW mixtures are commonly used as thermal fluids in heat exchange applications due to their attractive thermal features such as boiling and freezing points [29,30,31]. The NF sample of 0.5 L necessary for the experiment was prepared by two-step methods where appropriate quantities of nanoparticles were mixed with the BFs (EG/DW). The mixing of the NPs with the BF was performed by a magnetic stirrer for 20 min, followed by undertaking ultrasonication for another 25 min (amplitude of 60% and 40 kHz Frequency) using an ultrasonicator (Hielscher UP200Ht) which led to achieving good dispersion and stability of the NPs into the BF. The second use of the NFs is conducted after two months after their first use, and the same (aforementioned) dispersing process was repeated to each NF sample (magnetic stirring for 20 min and ultrasonication for 25 min). The Pictures of the NF samples in Figure 1 show the prepared NF samples’ new and recycled (reused) Al_2_O_3_ NFs before use in the PB experiments.

It can be noticed that there is no visible change in the colour or sedimentation before the first (new) and the second (recycled) uses of the NF samples, which leads to the fact that additional wastes (dust, rust, or residues from corrosion of cell and wire) have not been added from the PB system to the NF samples during the operation. It should be noted here that the system was newly built, and the components of the PB are new and were thoroughly cleaned before each experimental run.

### Density and UV Analysis for Stability and Concentrations of Nanoparticles

Thermophysical properties and characteristics of NFs are the key factors that affect their heat transfer performance and efficiency of single-phase and phase-change (e.g., boiling) based cooling systems. The operation of NFs in applications such as PB may cause a loss of NPs, which affects their features. The latter can be checked by analysing the density and UV results before the first and the second uses of the NF samples. For this purpose, the density of the samples is measured at room temperature by a Density Meter from Kem Kyoto Electronics, Kyoto, Japan. On the other hand, the UV-1280 spectrophotometer is used to evaluate the dispersion (stability) and concentration of the produced NFs. It should be mentioned that several experimental readings were taken for each NF sample, and the average values are reported. Moreover, the experimental apparatuses were validated, and a calibration procedure was followed to ensure the accuracy of the measurements. The measurements’ uncertainties for the density and absorbance (ABS) were 1.5% and 1.0% at the maximum, respectively.

**Figure 2:**
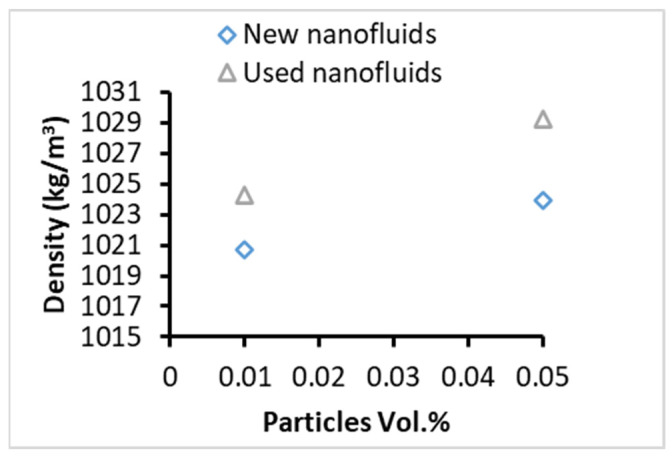
The density of the new and used Al_2_O_3_ NF samples.

The results presented in Figure 2 show an increase in the density of the NF after the use in PB up to 0.6% which refers to the slight loss of BF (15%EG/85%DW) in the evaporation. The latter also refers to the idea that NPs may not go with the vapour of the BF from the pool. On another hand, there was no noticeable deposition of nanoparticles at the bottom of the boiling cell during the measurements due to the random movement of NPs under the boiling in addition to the good stability achieved for the nanofluids during preparation. Furthermore, NPs may deposit on the surface of the heating wire, which cannot be observed visually due to the small area of the heating wire [32]. Nevertheless, based on research conducted on the boiling of nanofluids, it became certain that NPs deposit onto the boiling surface, forming micro/nano-structures that significantly influence the boiling procedure [25,33].

**Figure 3:**
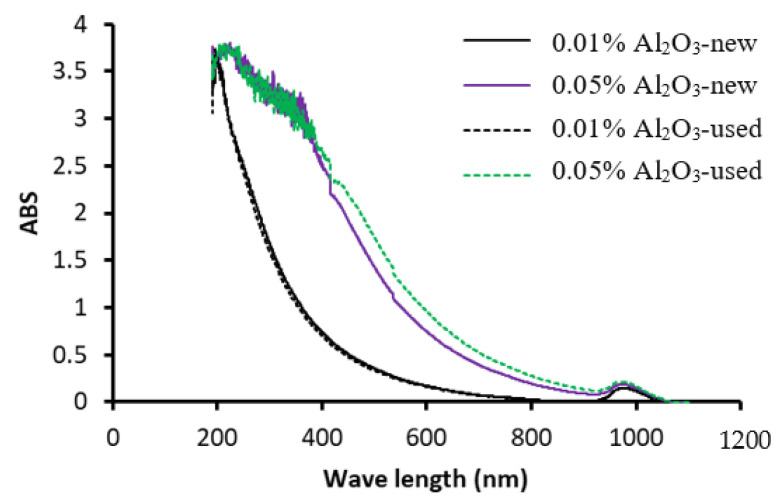
UV-Vis ABS spectra of the new and used NF samples.

UV spectrophotometer experiment is a common way to assess the dispersion of the NPs into the BF regarding concentration and colloidal stability [34,35,36]. Therefore, in this study, light waves of lengths between 190 nm and 1100 nm are selected for each NF sample. This allows for determining the change of the particles’ volume fraction suspended in the BF. The results of the UV absorbance of EG/DW mixtures based on the Al_2_O_3_ NFs of the two concentrations (0.01 and 0.05 vol.%) are displayed in Figure 3 for the two cases of the first use (new) and the second use (used). The results show the light absorbance curves for the several NF samples for the range of wavelengths between 190 nm and 1100 nm, and it appears how is the light absorbance’s value increased by increasing the particle concentration. Additionally, the UV results confirm the results of density presented in Figure 3 and show that reused (recycled) NFs present slightly higher absorbance (ABS) values for the PB system compared to new NFs. In this, the higher ABS values correspond to a higher particle concentration which can be explained by the slight loss of BF in the evaporation; thus, the second use of the NFs may contain slightly higher particle concentration.

However, the different results of light absorbance and subsequently the stability of Al_2_O_3_ NFs measured in different labs [37,38] can be due to the different preparation methods (mixing time, temperature, adding surfactants, etc.), particles size and concentrations, BF type, and storage conditions. In the current study, both the new and the used NF samples were in good stability condition after the preparation and during the experimental measurements.

## 3. Experimental Setup and Measurements

**Figure 4:**
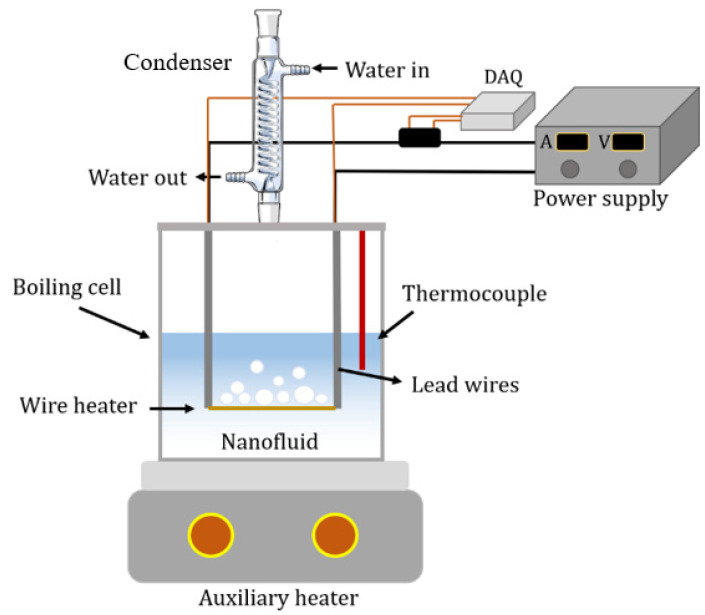
The pool boiling experimental setup (schematic).

In this study, the PB experimental setup was developed, and a schematic is presented in Figure 4. The experimental setup consists of an auxiliary heater at the bottom of the boiling cell to maintain the fluid in its saturation temperature, a vapor condenser provided by water flow (in and out) for cooling, a power supply (model of 6226 by PeakTech), and a data acquisition system (DAQ of USB-6008 from NI) associated with thermocouples K-type. The test section is a glass chamber that facilitates bubble visualisation and a temperature sensor to measure the liquid temperature. The heating element is a horizontal Nickel-Chromium (Ni-Cr) wire of 4.27 cm in length and 0.25 mm in diameter with a melting temperature of approximately 1400 °C.

**Figure 5:**
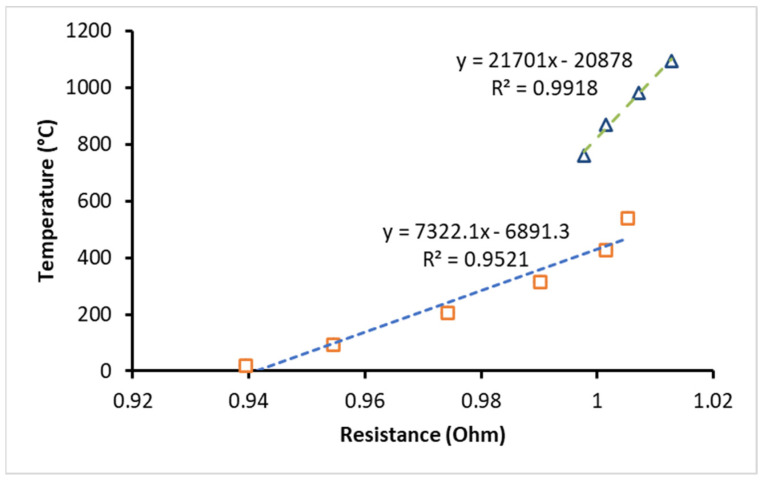
The calibration curve of temperature as a function of the resistance of the wire.

The BF and NF samples are first preheated in the glass chamber to maintain the sample at its saturation temperature. The voltage that is supplied from the power supply to the wire is increased in small steps, and the measurements of current and voltage are recorded. Then, the electrical resistance (*R*) of the heated wire is found from the obtained values of the current (*I*) and voltage (*V*) (i.e., *R* = *V*/*I*). The temperature of the wire (T_w_) is obtained by the relation between the electrical resistance of the wire and temperature in what is called “the calibration curve of the temperature-resistance”, determined through a similar methodology explained in the literature [39,40]. The calibration curve of the wire from this study is presented in Figure 5 for temperatures between 20 °C and 1100 °C and fitted linearly. As presented in the results, two linear correlations are obtained and used to determine the temperature of the wire, one from 0.94 Ohm to 0.99 Ohm, and another from 0.99 Ohm to 1.02 Ohm.

**Figure 6:**
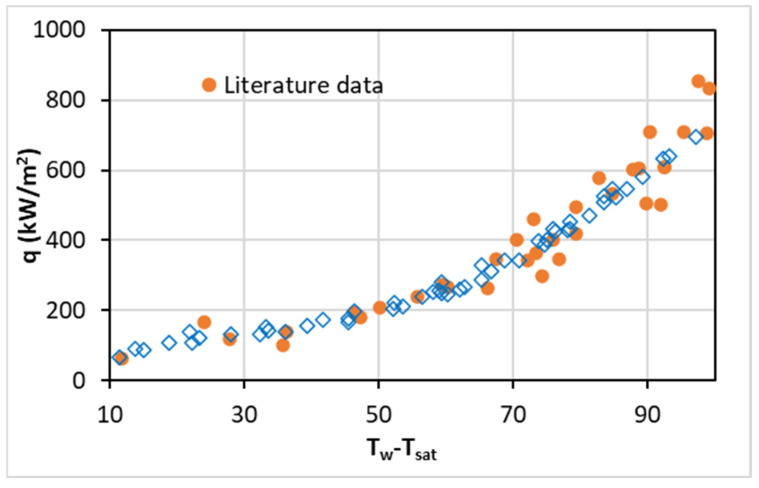
Comparison of PB experimental results of water with the data from the literature [39].

The experimental setup was tested first using DW and validated against the found data in the relevant literature [39] with similar operating conditions to ensure the reliability of the apparatus, as shown in Figure 6. The PB curve of DW (heat flux versus superheat) is determined and compared with the PB curve of DW from the literature [39] in the range of nucleate boiling for similar characteristics of the PB setup and the operation conditions.

Figure 6 demonstrates that the measured results of the established PB system are in good agreement with the data from a relevant study from the literature, and the good reliability of the setup is confirmed.

On other hand, the trend of the obtained boiling curve (including the nucleate boiling at high superheat values) is found to be similar to other similar boiling studies with nanofluids and their base fluids, such as the study by Sarafraz and Hormozi [41] for dilute CuO/water nanofluid and a study by Song et al. [42] for SiC/water nanofluid in pool boiling. It is to be mentioned that the user type of the heating element (plate, wire, etc.) and the modifications of the heating surfaces (under different dimensions from macroscale to nanoscale and using different materials) can change the effective heat transfer area, the density of nucleation site, wettability and other factors that affect the profile of the nucleate boiling regime [25,43]. Furthermore, the operating conditions of the PB (e.g., saturation pressure) and the properties of the used fluid may lead to different boiling results even for the same type of nanofluids [28,44].

After the validation of the setup, the BF (EG/DW) and the NF samples were tested in the PB system. Therefore, the boiling curve of the BF and NFs can be obtained by plotting the heat flux (*q*) as a function of the wall superheat (T_w_ − T_sat_), where T_w_ is the wire heater temperature and T_sat_ is the saturation temperature of the sample fluids, measured by the thermocouple. The CHF can be obtained by analysing the obtained PB curve. The heat flux is calculated from the following expression:

(1) $$q=\frac{VI}{\pi dL}$$

where *V* is voltage, *I* is current, *d* is the wire diameter, and *L* is the wire length. Additionally, the HTC of the NF samples can be calculated from the special form of Newton’s cooling law which is as follows:

(2) $$h=\frac{q}{T_{w}-T_{s}}$$

## 4. Results and Discussion

The heat transfer features of the NFs through a PB heat transfer system are investigated, taking into account the conditions of the second use (after recycling) of the NFs and their shelf life.

### Pool Boiling Results

**Figure 7:**
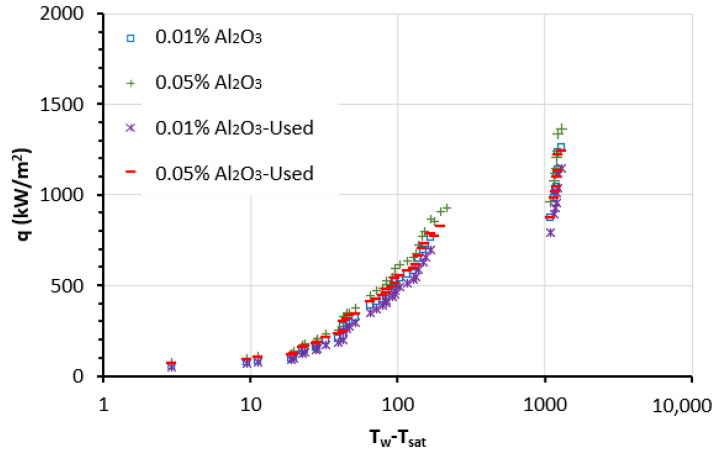
Pool boiling curve for EG/DW mixture-based Al_2_O_3_ NFs.

The experiments for the NF samples in the PB were conducted, and the data were collected to determine the heat flux (q) and the wall superheat (T_w_ − T_sat_) for each sample. The boiling curves for Al_2_O_3_ NFs are presented in Figure 7 for the two concentrations of 0.01 and 0.05 vol.% of NPs and for the new and reused NFs. The results indicate that the wire superheat (T_w_ − T_sat_) of the Al_2_O_3_ NFs at the primary boiling phase (natural convection) is lower than the BF for the same heat flux value. Additionally, adding the NPs to the BF pulls the boiling curve to the left, confirming early nucleation and reaching of CHF. Furthermore, the CHF of the operated NFs is greater than that of the BF and increases with the rise of NPs concentration. The results of the new NFs present an enhancement in CHF (in comparison with BF) of about 60% at 0.05 vol.% particle concentration and 41% enhancement at 0.01 vol.%. While the results of the second use (reused) of the NFs in comparison with BF indicate an enhancement in CHF of about 35% at 0.05 vol.% particle concentration and a 21% enhancement at 0.01 vol.%, it is clear that the recycled (used) NFs have a slightly lower enhancement of CHF when compared to the new Al_2_O_3_ NFs with an average deviation of about 23%.

**Figure 8:**
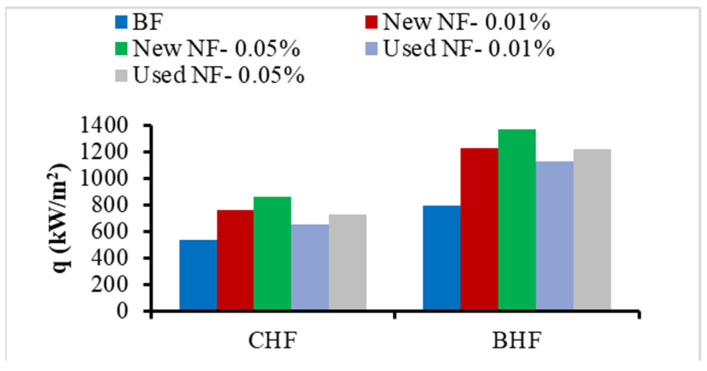
CHF and BHF values for the EG/DW mixture-based Al_2_O_3_ NFs.

On another hand, the values of the burnout heat flux (BHF) can be obtained for each NF from the boiling curves and compared with CHF values, as presented in Figure 8. The results show enhancements of the BHF of about 73% at 0.05 vol.% and 54% at 0.01 vol.% for the new NFs, and 53% at 0.05 vol.% and 43% at 0.01 vol.% for the reused NFs. Thus, the BHF of the NFs is enhanced in a similar trend with CHF by adding the NPs, and, as anticipated, higher values are obtained for the BHF as well as better enhancement compared to BF.

**Figure 9:**
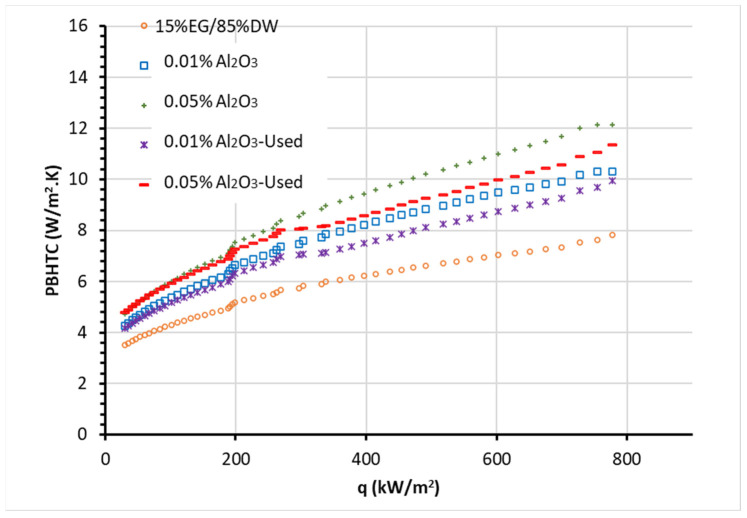
PBHTC of EG/DW mixture-based new and used Al_2_O_3_ NFs as a function of heat flux.

Furthermore, the results of PBHTC of the NF samples and BF are presented in Figure 9 for the nucleate boiling regime. The results report an increase in the PBHTC values with rising heat flux and NP concentration, with increasing enhancements of PBHTC compared to the BF. The maximum PBHTC enhancements of Al_2_O_3_ NFs at 0.01 vol.% and 0.05 vol.% are approximately 29% and 48%, respectively, for the newly prepared NFs compared to the BF. While the second use (after recycling) of the NFs presented around 21% and 35% of PBHTC, respectively, at 0.01 vol.% and 0.05 vol.% enhancements in comparison with the BF.

Therefore, the experimental findings as presented in Figure 7, Figure 8 and Figure 9 prove that the combination of NPs (such as Al_2_O_3_) having higher thermal properties (e.g., thermal conductivity) and a thermal fluid (i.e., DW/EG mixture in this case) enhances the boiling (pool) heat transfer performance. In addition, the results described the raise in NPs’ concentrations from 0.01 to 0.05 led to higher enhancement in heat transfer characteristics in PB. It should be also noted that the NF samples in the PB are at high temperatures (at saturation), resulting in an even higher thermal conduction property and lesser viscosity of the samples. On the other hand, the change of thermophysical characteristics, including density and other parameters, such as the surface tension of the NFs that happen over time, can further impact the heat transfer in PB. These could also be the reasons for the better feature of the heat transfer for new Al_2_O_3_ NFs than for the recycled NFs. Where the newly prepared NFs show higher boiling heat transfer enhancements when compared with reused (recycled) NFs with a deviation of up to 20%. Additionally, this deviation decreases with the reduction of concentrations of particles (Figure 7, Figure 8 and Figure 9) as the lower concentration, the smaller the number density of NP leading to relatively less deposition of NPs on the heater surface for both NFs cases.

Apart from the properties and characteristics of NFs, it is still not possible to identify the main mechanisms for the enhanced boiling heat transfer features and performance of NFs. So far, it is known that there is an exchange interaction between the NPs and BF that affects their movements and plays an important role in the heat transfer of NFs [45]. In this, a study by Wang et al. [46] has mentioned important mechanisms of the NPs in NF mediums, such as the chaotic convection caused by the electrophoretic and thermophoretic motion of NPs that affect the heat transfer performance of NFs. Moreover, the effect of NPs deposition on the surface of the heating wire in a PB can contribute largely to such improvement in CHF, PBHTC, and BHF [20,21,47]. The bumps and pits that are caused by the particle’s deposition on the surface of the wire heater increase the bubble nucleation and vaporisation cores [27]. Additionally, the modification of the bubble dynamics in the PB caused by the nanoparticles has a significant influence on the heat transfer performance of the NFs in a PB [48]. Furthermore, the good thermal conductivity of the Al_2_O_3_ particles and their motion enhances the heat exchange between the fluid and the wire; thus, the bubbles are generated earlier, delaying the arrival of CHF and BHF. In the case of BF (conventional thermal fluids) used in PB, the number of bubble nucleation sites is presumed to be less than that of NFs; thus, the bubble formation rate is higher than the departure rate and bubble coalescence happens on the wire surface, reaching the CHF and BHF. The latter explains the higher enhanced values of CHF, PBHTC, and BHF of NFs when compared with the related BF in PB application. Furthermore, it should be mentioned that some factors in NFs are responsible for their significant thermal enhancement of the PB process (e.g., the sedimentation layer of nanoparticles on the wire surface and the surface oxidation affect the capillary wicking property); thus, it may reduce the nucleation site area for each bubble [22]. Moreover, the sedimentation of nanoparticles and bubble movements of the heating surface can vary because of different factors such as the type and concentration of the nanoparticles as well as the orientation of the heater surface for non-cylindrical or spherical heater geometry, thus affecting the heat transfer performance [26].

## 5. Conclusions

In this study, the thermal characteristics of 15%EG/85%DW (*v*/*v*) based new and recycled (used) Al_2_O_3_ NFs with two different concentrations (0.01 and 0.05 vol.%) in the PB system were experimentally investigated. The stability and physical characteristics of both NFs are evaluated. A pool boiling experimental setup was built which was calibrated as well as validated with DW. The experimental results regarding the heat flux as a function of the superheat were collected and the boiling curve was prepared. Then, the CHF, PBHTC, and BHF values were determined at each particle’s concentration for the new and the recycled NF samples. It was found that there is a good enhancement in CHF up to 60% and an enhancement in BHF up to 54% for the new Al_2_O_3_ NFs of 0.05 vol.% concentration. Additionally, the PBHTC was found to increase with increasing the heat flux and NP concentration up to 48% for the newly prepared NFs compared to the BF. Whereas the recycled nanofluids showed a lower enhancement (by about 20%) of heat transfer when compared with the new NF samples. Nevertheless, the used NF samples still exhibit considerably higher boiling heat transfer performance compared to BFs, which is of great importance for their recycling in the same applications. For the first time, it is demonstrated that used Al_2_O_3_ NF samples have great potential to be recycled in the same systems or processes as this study’s pool boiling one.

## Data Availability

Not applicable.

## Nomenclature

Al_2_O_3_	Alumina
BF	Base fluid
BHF	Burnout heat flux
CHF	Critical heat flux
DW	Distilled water
d	Wire diameter
EG	Ethylene glycol
PB	Pool boiling
PBHTC	Pool boiling heat transfer coefficient
I	Current
L	Wire length
NF	Nanofluid
NP	Nanoparticles
q	Heat flux
R	Electrical resistance
T_w_	Wire surface temperature
T_sat_	Saturation temperature of the fluid
V	Voltage
